# Improving Colorectal Cancer Screening and Risk Assessment through Predictive Modeling on Medical Images and Records

**DOI:** 10.1016/j.ajpath.2025.09.016

**Published:** 2025-10-16

**Authors:** Shuai Jiang, Christina Robinson, Joseph Anderson, William Hisey, Lynn Butterly, Arief Suriawinata, Saeed Hassanpour

**Affiliations:** ∗ Department of Biomedical Data Science, Geisel School of Medicine at Dartmouth, Hanover, New Hampshire; † Department of Gastroenterology and Hepatology, Dartmouth-Hitchcock Medical Center, Lebanon, New Hampshire; ‡ New Hampshire Colonoscopy Registry, Lebanon, New Hampshire; § Department of Medicine, Dartmouth-Hitchcock Medical Center, Lebanon, New Hampshire; ¶ White River Junction VA Medical Center, Hartford, Vermont; ‖ Department of Pathology and Laboratory Medicine, Dartmouth-Hitchcock Medical Center, Lebanon, New Hampshire; ∗∗ Department of Epidemiology, Geisel School of Medicine at Dartmouth, Hanover, New Hampshire; †† Department of Computer Science, Dartmouth College, Hanover, New Hampshire

## Abstract

Colonoscopy screening effectively identifies and removes polyps before they progress to colorectal cancer (CRC), but current follow-up guidelines rely primarily on histopathologic features, overlooking other important CRC risk factors. Variability in polyp characterization among pathologists also hinders consistent surveillance decisions. Advances in digital pathology and deep learning enable the integration of pathology slides and medical records for more accurate progression risk prediction. Using data from the New Hampshire Colonoscopy Registry, including longitudinal follow-up, a transformer-based model for histopathology image analysis was adapted to predict 5-year progression risk. Multi-modal fusion strategies were further explored to combine clinical records with deep learning–derived image features. Training the model to predict intermediate clinical variables improved 5-year progression risk prediction [area under the receiver-operating characteristic curve (AUC), 0.630] compared with direct prediction (AUC, 0.615; *P* = 0.013). Integrating whole-slide imaging–based model predictions with nonimaging features further improved performance (AUC, 0.672), significantly outperforming the nonimaging-only approach (AUC, 0.666; *P* = 0.002). These results highlight the value of integrating diverse data modalities with computational methods to enhance progression risk stratification.

It is estimated that 52,550 lives were lost in 2023 due to colorectal cancer (CRC) in the United States, ranking CRC as the second-highest cancer mortality rate after lung cancer. Overall, 4.3% (1 in 23) of men and 3.9% (1 in 26) of women will be diagnosed with CRC in their lifetime.^1^ However, CRC can be prevented through regular screening procedures,^2^^,^^3^ as almost all CRC arises from colorectal polyps [thereafter referred to as “polyp(s)”].^4^ Meanwhile, nearly one-half of Western adults will have a polyp in their lifetime, and one-tenth of these cases will progress to cancer.^5^ Fortunately, it usually takes several years for these polyps to progress, leaving a window for removal and CRC prevention.

Currently in the United States, it is recommended that all average-risk adults undergo CRC screening by the age of 45 years; patients at increased risk, such as those with a family history of CRC in a close relative, need to start screening earlier. Colonoscopy screening is associated with a 40% to 67% reduction in the risk of death from CRC.^6^^,^^7^

There are multiple “direct-visualization” screening methods to detect polyps, such as colonoscopy, sigmoidoscopy, and virtual colonoscopy. Among these, colonoscopy has become the most common screening test in the United States.^8^ More than 15 million colonoscopies are performed in the United States each year.^9^^,^^10^ In 2021, it was estimated that 63.1% of U.S. adults were up to date with colonoscopy screening, and it is expected that CRC screening will increase to cover 74.4% of Americans aged 50 to 75 years by 2030.^8^

In 2020, the U.S. Multi-Society Task Force on CRC issued updated guidelines on CRC surveillance after colonoscopy screening and polypectomy.^11^, ^12^, ^13^ The Task Force recognizes a range of CRC risk classifications for patients undergoing colonoscopies. The timing of the recommended follow-up colonoscopy depends on these categories: <5 years for high-risk patients and 5 to 10 years for low-risk patients. Of note, according to the Task Force guidelines, risk and follow-up recommendations for patients undergoing colonoscopy depend only on histopathologic characterization (ie, type), number, size, and location of polyps detected in previous colonoscopies. Therefore, the current screening and surveillance guidelines are limited and do not take into account many other CRC risk factors such as age, race, body mass index, smoking/alcohol use, activity level, diet, and family history.^14^, ^15^, ^16^, ^17^, ^18^, ^19^, ^20^, ^21^, ^22^, ^23^ Incorporating these relevant factors into CRC risk assessment aims to capture a comprehensive context to provide patients with a more accurate and efficient surveillance plan.

Another gap in CRC risk stratification arises from the microscopic examination of polyps. Although the recommended intervals between surveillance procedures depend on histopathologic analysis,^12^ accurate reading can be a challenging task^4^; currently, there is a significant amount of variability among pathologists in how they characterize and diagnose colorectal polyps.^24^, ^25^, ^26^, ^27^, ^28^, ^29^, ^30^, ^31^, ^32^, ^33^, ^34^, ^35^ For instance, sessile serrated polyps, which can develop dysplasia and progress to CRC, are difficult to differentiate histologically from hyperplastic polyps.^36^, ^37^, ^38^ The accurate characterization and differentiation of polyps can help patients to receive appropriate follow-up surveillance and to reduce unnecessary additional screenings, health care costs, and stress.

With the recent expansion of whole-slide digital scanning, high-throughput tissue banks, and archiving of digitized histologic studies, the field of digital pathology is primed to benefit significantly from deep learning models. A major advantage of deep learning for histopathologic image analysis is eliminating the need to design application-specific, handcrafted features for training the model; thus, it can be applied to various tasks, including nucleus detection,^39^, ^40^, ^41^ tumor classification,^42^, ^43^, ^44^, ^45^, ^46^, ^47^, ^48^, ^49^ and patient outcome prediction.^50^, ^51^, ^52^, ^53^ Because transformer models reshape the landscape of medical image analysis, they have been applied to CRC-related tasks such as CRC segmentation^54^ and colorectal polyp classification.^55^ In a substantial multicenter study, transformer models were observed to outperform convolutional neural network–based methods in predicting microsatellite instability.^56^

Histopathologic information provides crucial insights into tumor characteristics; however, for a comprehensive analysis, the inclusion of nonimage features is imperative. Multi-modal fusion is a key strategy for combining information from diverse modalities to augment prediction accuracy. These fusion techniques are broadly categorized into early fusion and late fusion. Early fusion involves concatenating features from each modality and training a unified model, whereas late fusion entails training individual models and combining their decisions.^57^ Numerous studies adopt the approach of concatenating features from different modalities to construct a unified patient representation.^51^^,^^58^, ^59^, ^60^, ^61^ Although feature-level fusion has the potential to capture intricate correlations among features, its flexibility may elevate the risk of overfitting. Conversely, some studies have shown that simpler decision-level fusion techniques outperform feature-level fusion in certain scenarios.^62^^,^^63^

A key requirement for developing accurate deep learning methods is the access to large data sets for model training. Despite the rapid advancements in deep learning technology and computational capabilities, no major efforts have been made to curate large, open-access, and high-quality annotated images for polyp analysis. The lack of “big data” in this domain makes it virtually impossible for existing or new deep learning architectures to be developed for polyp histopathologic characterization. Data collected from the New Hampshire Colonoscopy Registry (NHCR) were used in the current study for model development and evaluation.

The overall objective of the current study was to develop and evaluate a novel, accurate, and automatic deep learning method to analyze clinicopathologic findings associated with colorectal polyps for CRC prevention by leveraging both imaging and medical record information. In a previous study,^64^ a transformer-based pretraining and fine-tuning pipeline, MaskHIT, was developed for medical image analysis by this group. In this study, the MaskHIT model was adapted for automatic, accurate, and interpretable polyp characterization on histology images. In addition, different fine-tuning strategies (direct versus guided) and various multi-modal fusion techniques were explored to improve progression risk assessment based on both imaging and nonimaging data. The improved accuracy in future progression risk prediction was anticipated to benefit colonoscopy participants in making more informed follow-up decisions.

## Materials and Methods

### Study Population

The NHCR is a National Cancer Institute–funded, statewide registry that contains comprehensive longitudinal colonoscopy information from nearly all endoscopy sites in New Hampshire since 2004. It includes patient risk factors, such as: age; sex; personal and family history of polyps or CRC; weight; height; smoking status; alcohol consumption; endoscopy history; polyp sizes, locations, numbers, and treatment; pathology reports; follow-up recommendations; and follow-up outcomes.^65^^,^^66^ These data are extracted through a rigorous data collection effort from 31 participating practices in addition to questionnaire responses from patients. The NHCR is unique in the United States in terms of its detailed, population-based, longitudinal, and comprehensive data set. Dartmouth Hitchcock Medical Center (DHMC), a tertiary academic medical center in Lebanon, New Hampshire, has been participating in the NHCR from its start in 2004, and, among patients who have their information recorded in the NHCR, >30,000 are DHMC patients. The histology slides of these DHMC patients are stored at the Department of Pathology and Laboratory Medicine at DHMC and were available for the current study.

The sources of data in this project include the NHCR^67^^,^^68^ and pathology slides from the DHMC. A total of 2598 patients who underwent colonoscopy at the DHMC from 2004 to 2018 without a CRC diagnosis at the index visit, and who had digitized polyp slides from their baseline colonoscopies with CRC status reassessed after 5 years, were included in this study. After excluding 205 patients with missing clinical data, 2393 patients were included in the training and evaluation of the proposed models. This data set encompasses hematoxylin and eosin–stained whole-slide images (WSIs) with various types of polyps, along with patients’ clinical records from the NHCR.

### Outcome

The NHCR and DHMC follow-up medical records are used to identify patients at high risk of CRC and to build the progression risk reference standard labels for patients. Based on polyp recurrence rate, CRC progression time, and the recommended frequency for follow-up colonoscopies,^11^^,^^12^ patients in this study who developed CRC, advanced adenomatous polyps, or serrated polyps with dysplasia in the 5 years after screening were considered as high risk; patients without those developments within 5 years were considered low risk. Advanced adenomatous polyps include polyps ≥1 cm, with villous components (tubulovillous adenoma/villous adenoma), or with high-grade dysplasia.^69^ Advanced adenomas and serrated polyps ≥1 cm or with dysplasia are known as surrogates for CRC and are widely used as indicators of high progression risk.^70^, ^71^, ^72^, ^73^, ^74^ The 5-year risk window is chosen to maximize the clinical utility, based on the use case in this project and the current guidelines for follow-up colonoscopy intervals.^11^^,^^12^

### Clinical Features and Image Data

Under the review and approval of the Committee for the Protection of Human Subjects, the following information from the NHCR database was extracted: i) identifiers of patients with tissue removed during colonoscopy, including the pathology Case-ID, used to locate tissue slides and access WSIs; ii) relevant medical information; iii) types, numbers, and sizes of polyps identified in the index colonoscopy examination; and iv) outcome determination within 5 years after the index colonoscopy.

The medical information was collected from NHCR Procedure Forms, completed by endoscopists or endoscopy nurses at participating sites, and patient questionnaire responses. The NHCR database covers a comprehensive list of CRC risk factors based on peer-reviewed publications.^65^^,^^66^ The variables extracted from the NHCR database are summarized in five categories, as shown in Supplemental Table S1.

Hematoxylin and eosin–stained WSIs, scanned at DHMC (Aperio AT2; Leica Biosystems), were processed by using the MaskHIT pipeline. Briefly, a color thresholding technique was used to create tissue masks. Non-overlapping patches of size 224 μm × 224 μm (ie, 448 × 448 pixels) at a 20× magnification level (0.5 μm per pixel) were extracted, along with their positions on the WSI.

### Risk Prediction Using WSIs

The MaskHIT architecture is used for predicting 5-year progression risk using WSIs. MaskHIT can effectively model the relative positional information of patches on a large region from the WSI. In the pretraining phase, the Masked AutoEncoder technique was used, first randomly masking out a portion of patches from the sampled region, then using the output from the transformer model to restore the feature representations of those masked locations. This process helps the model capture relationships between different patches and their histopathologic features and understand the context. The original MaskHIT model was pretrained using >10 cancer types from The Cancer Genome Atlas database (*https://tcga-data.nci.nih.gov/docs/publications/tcga*, last accessed October 30, 2025). The MaskHIT model achieved improved performance in cancer survival prediction and cancer subtype classification tasks compared with state-of-the-art models.

**Figure 1:**
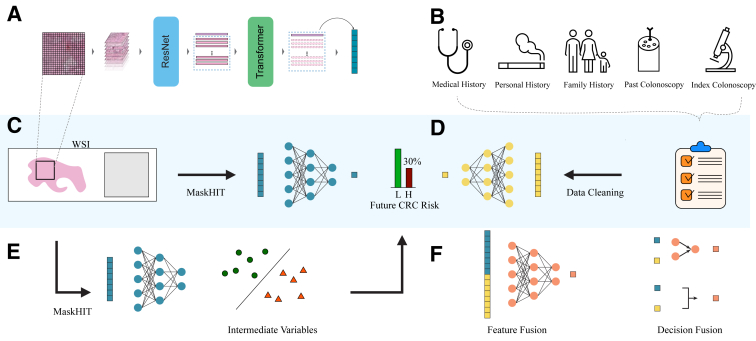
Technical overview. **A:** Illustration of the MaskHIT pipeline. **B:** Data sources of medical records. **C:** Direct prediction of 5-year progression risk using whole-slide images (WSIs). **D:** Prediction of 5-year progression risk using medical records. **E:** Guided attention approach that fine-tunes MaskHIT first for intermediate variables and then for the 5-year progression risk. **F:** Different fusion methods. CRC, colorectal cancer; H, high CRC risk; L, low CRC risk.

The workflow of the MaskHIT model involves the extraction of square regions, comprising up to 400 patches, from WSIs. Subsequently, a ResNet model, pretrained on ImageNet data,^75^ is used for feature extraction. The location information of the patches, along with the extracted features, is then fed into a transformer model, which comprises eight attention heads and 12 attention layers. The output of the transformer model yields a class token, serving as a representation of the entire region. Multiple regions can be sampled from a single WSI concurrently, and the class tokens are averaged to generate a global summarization of the WSI. This global summarization is then used for risk classification through a linear projection layer (Figure 1, A and C).

To tailor the MaskHIT model pretrained on The Cancer Genome Atlas database to the specific context of polyp analysis, an additional pretraining phase for 200 epochs was conducted, using the same pretraining methodology as previously described.^76^ During the fine-tuning stage, the model randomly sampled four nonoverlapping regions from each patient. Each region comprised up to 400 patches, each of size 448 × 448 pixels at a 20× magnification level. To mitigate computational costs of fine-tuning, 25% of the patches were randomly sampled from each region. During the evaluation phase, a maximum of 64 regions were sampled from each patient across all slides for that patient to estimate progression risk.

Beyond the direct risk prediction using WSIs, an alternative training approach, named “guided prediction” (Figure 1E), was explored. In this procedure, a MaskHIT model was initially fine-tuned to predict intermediate variables derived from patients’ pathology reports at the index visit. Subsequently, this model was used to predict patients’ future progression risk. Two strategies were compared: i) freezing the weights of the transformer model and exclusively fine-tuning the last linear projection layer for outcome prediction; and ii) fine-tuning both the transformer model weights and the last linear projection layer. The guided prediction approach uses intermediate variables only during the training procedure to assist the MaskHIT model in focusing on more relevant regions in the WSIs.

### Combination of Clinical and Image Histopathologic Information

The nonimaging variables (Figure 1B) underwent preprocessing, in which continuous variables were standardized and categorical variables were one-hot encoded, resulting in a feature vector of dimension 69. Missing values were imputed by replacing them with either the average value for continuous variables or the most common class for categorical variables.

Common approaches for risk prediction, such as penalized logistic regression, multi-layer perceptron, and random forest, were explored for modeling nonimaging variables. Each approach was evaluated through cross-validation on a subset withheld from the training data to select the optimal architecture for modeling nonimaging variables (Figure 1D).

Various strategies for integrating nonimaging variables with WSI features were investigated, including feature-level aggregation and decision-level aggregation (Figure 1F). Feature-level aggregation, an early fusion technique, concatenated nonimaging features with those extracted from the transformer output of WSIs. Subsequently, patient outcomes were predicted by using multiple layers of linear projections. In decision-level fusion, predicted risk probabilities from nonimaging variables and WSIs were combined either through averaging or by assigning different weights to each component.

### Evaluation

In this study, 25% of the data set (ie, slides and records for 600 patients) was held out as the test set for evaluation of the developed methods, and the remaining 75% was used as the training set. A fivefold cross-validation was conducted on the training data set for hyperparameter tuning. The area under the receiver-operating characteristic curve (AUC) was used to assess the model’s performance. To ensure a more robust estimate of model performance, the train/test splitting process was repeated 10 times, and the average performance along with the SD on the test splits were reported. Paired *t*-tests across repeated experiments were used to calculate the statistical significance (*P* < 0.05) of the compared methods.

### Model Interpretation and Visualization

A significant limitation of current deep learning methods is their black-box nature, in which the focus is primarily on the efficacy of the final results, with little attention given to providing clear explanations or evidence of the factors that contribute to these outcomes. To address this issue and gain a deeper understanding of the pertinent regions on WSI influencing risk predictions, the difference in attention scores between the pretrained transformer model and the transformer model fine-tuned for outcome prediction was computed. These attention score differences were then color-coded and overlaid onto the WSIs, enabling insights into the shift in model attention for each specific outcome prediction task.

For the multi-modal fusion model that integrates nonimaging information with the WSI, interpretability was enhanced by calculating Shapley values for both nonimaging features and WSI risk predictions. These Shapley values were aggregated across repeated experiments, and the average scores were plotted for visualization, providing a transparent depiction of the contributions of each feature to the model’s predictions.

## Results

### Description of Study Population

A description of the demographic features of the study population is presented in Supplemental Table S2. Of 2393 patients, 1994 (83.3%) remained in the low-risk category after 5 years, whereas 399 (16.7%) developed high-risk findings. The patients who developed high progression risk in 5 years were significantly older than those who remained in the low progression risk category (62.0 years versus 58.7 years; *P* < 0.001) and were more likely to be male (60.7% versus 51.9%; *P* = 0.002). Much of the study population was non-Hispanic White, and the distribution of race and ethnicity did not differ by risk group. Descriptions of other groups of variables are provided in Supplemental Tables S3 to S7.

### Risk Prediction Using WSIs

In the direct prediction of 5-year progression risk using WSIs, the MaskHIT model attained an average AUC of 0.615. Multiple intermediate variables were evaluated, including size and number of adenomas, size and number of serrated lesions, most advanced serrated lesion, most advanced adenoma, and all of them combined (Table 1).

**Table 1:** Comparison of AUC (SD) for Direct Prediction Versus Guided Attention Prediction of 5-Year Progression Risk Using WSIs

Fine-tuning strategy	Intermediate variable	WSI →intermediate	Intermediate →risk	WSI →risk	
				Freeze transformer	Fine-tune transformer
Direct prediction	None	–	–	–	0.615 (0.016)
Guided prediction	Largest known adenoma size	0.889 (0.004)	0.593 (0.026)	0.625 (0.016)	0.626 (0.016)∗
	No. of adenomas	0.800 (0.007)	0.643 (0.029)	0.624 (0.014)	0.625 (0.016)
	Most advanced adenoma	0.902 (0.004)	0.550 (0.024)	0.618 (0.018)	0.620 (0.016)
	Largest known serrated lesion size	0.897 (0.004)	0.511 (0.034)	0.623 (0.018)	0.629 (0.016)∗
	No. of serrated lesions	0.864 (0.002)	0.482 (0.037)	0.614 (0.028)	0.622 (0.014)
	Most advanced serrated	0.927 (0.007)	0.546 (0.019)	0.625 (0.018)	0.625 (0.018)∗
	All the above variables	–	0.651 (0.027)	0.622 (0.015)	0.630 (0.016)∗

Most advanced adenoma (categorical variable with levels): no adenoma, tubular adenoma, tubulovillous adenoma, villous adenoma. Most advanced serrated (categorical variable with levels): no serrated polyp, hyperplastic polyp, sessile serrated polyp without dysplasia, sessile serrated polyp with dysplasia, traditional serrated adenoma.

AUC, area under the receiver-operating characteristic curve; WSI, whole-slide image.

∗ *P* < 0.05. *P* values were calculated by comparing the guided prediction results with the direct prediction using a paired *t*-test.

MaskHIT exhibited robust predictive performance for various intermediate variables, with notable AUC values. The highest AUC was achieved when predicting the most advanced serrated lesion (AUC, 0.927 ± 0.007), followed closely by predictions for the most advanced adenoma (AUC, 0.902 ± 0.004). The prediction of the number of adenomas found in colonoscopy yielded a slightly lower AUC at 0.800 ± 0.007. Overall, MaskHIT exhibited effective predictive capabilities across a range of intermediate variables.

These colonoscopy findings can predict 5-year progression risk with various performances (Table 1). Measurements of the size and number of adenomas were better at predicting 5-year progression risk than measurements of serrated lesions. The best predictor among them was number of adenomas (AUC, 0.643 ± 0.029), whereas the AUCs obtained using measurements of serrated lesions were no better than a random guess. Measurements including most advanced adenoma or serrated lesion, although still contributing to prediction, achieved an AUC of approximately 0.55 in forecasting 5-year progression risk.

Using the guided attention approach, in which the MaskHIT model was initially fine-tuned for intermediate variables and subsequently fine-tuned for 5-year progression risk prediction, most intermediate variables exhibited an enhancement in outcome prediction performance. The best performance was observed when using the size of the largest known serrated lesion as the intermediate variable, achieving an AUC of 0.629 ± 0.016, although this variable itself cannot predict 5-year progression risk better than a random guess. When incorporating all colonoscopy variables as intermediate variables, the MaskHIT model achieved an average AUC of 0.622 ± 0.015 when the transformer backend was frozen. Further fine-tuning the transformer backend for risk prediction resulted in an average AUC of 0.630 ± 0.016), representing a statistically significant improvement compared with the direct prediction approach.

### Risk Prediction Using Medical Records

The performance comparison of L2 penalized logistic regression, random forest, and neural network models for predicting 5-year progression risk using nonimaging variables is presented in Table 2. There was no clear winner among these three prediction methods. Variables extracted from the index colonoscopy examinations displayed the best performance in predicting 5-year progression risk (AUC, 0.654–0.662), followed by personal history–related variables (AUC, 0.588–0.593). Previous colonoscopy history variables showed limited capability to predict 5-year progression risk AUC with an AUC of 0.514 to 0.547. However, medical and family history variables did not seem to contribute significantly to progression risk prediction.

**Table 2:** Risk Prediction Performance Using Nonimaging Variables, Reported as AUC (SD)

Category	Random forest	L2-logistic	Neural network
Personal history	0.588 (0.023)	0.592 (0.034)	0.593 (0.028)
Medical history	0.510 (0.017)	0.509 (0.021)	0.505 (0.016)
Family history	0.519 (0.020)	0.511 (0.023)	0.514 (0.024)
Previous colonoscopy	0.547 (0.015)	0.514 (0.027)	0.530 (0.025)
Index colonoscopy	0.654 (0.034)	0.662 (0.030)	0.661 (0.029)

AUC, area under the receiver-operating characteristic curve.

#### Multi-Modal Prediction

Table 3 compares different fusion strategies, including decision-level average and weighting, and the incorporation of WSI-predicted risk score and WSI-extracted features with nonimaging features. The results were stratified according to the strategy of fine-tuning the MaskHIT model for 5-year risk prediction. In both cases, the best multi-modal fusion performance was achieved when using the weighted average of the independent probabilities from WSIs and the nonimaging information (direction prediction training AUC, 0.669 ± 0.019; guided prediction training AUC, 0.672 ± 0.019). On average, decision-level fusion not only provides improved performance but also lower variation across the 10 repeated experiments compared with feature-level fusion.

**Table 3:** Comparison of Fusion Techniques for Risk Prediction, Reported as AUC (SD)

Fusion method	Direct	Guided
Decision average	0.665 (0.011)	0.670 (0.016)
Decision weighted	**0.669 (0.019)**	**0.672 (0.019)**
WSI decision + nonimaging features	0.661 (0.031)	0.668 (0.022)
WSI features + nonimaging features	0.664 (0.024)	0.668 (0.024)

Nonimaging features include index colonoscopy findings and additional medical records. Bold indicates the best performance of each column.

AUC, area under the receiver-operating characteristic curve.

In Table 4, the 5-year progression risk prediction performances resulting from diverse combinations of medical records, colonoscopy findings, and WSI risk predictions are presented. In this experiment, weighted decisions were used to fuse WSI-based predicted probabilities with the predicted probability from nonimaging features. Using medical record variables (all nonimaging variables excluding the index colonoscopy findings) or colonoscopy-only findings yielded an average AUC of 0.592 ± 0.032 and 0.662 ± 0.030, respectively, whereas the combination of both showed some improvements (AUC, 0.666 ± 0.023).

**Table 4:** Comparison of Input Modalities for Progression Risk Prediction, Reported as AUC Performance (SD)

Input modalities	AUC	Accuracy	F1	Precision	Recall
Medical records	0.592 (0.032)	0.570 (0.025)	0.305 (0.032)	0.208 (0.021)	0.578 (0.079)
Colonoscopy	0.662 (0.030)	**0.659 (0.023)**	0.354 (0.027)	**0.257 (0.020)**	0.571 (0.059)
Colonoscopy + medical	0.666 (0.023)	0.634 (0.017)	0.355 (0.024)	0.250 (0.016)	0.615 (0.056)
Colonoscopy + WSI	0.668 (0.025)	0.656 (0.021)	0.352 (0.022)	0.255 (0.013)	0.570 (0.068)
Colonoscopy + medical + WSI	**0.672 (0.019)**	0.638 (0.020)	**0.360 (0.018)**	0.254 (0.013)	**0.622 (0.054)**

Medical records includes all nonimaging variables excluding findings from index colonoscopy. Colonoscopy includes findings from index colonoscopy. Bold indicates the best performance in each column.

AUC, area under the receiver-operating characteristic curve; WSI, whole-slide image–predicted risk score.

Incorporating WSI-predicted risk scores led to noteworthy improvements. Specifically, the combination of colonoscopy findings with WSI risk scores presented an average AUC value of 0.668 ± 0.025, whereas the combination of all three modalities further improved the AUC to 0.672 ± 0.019; both improvements were statistically significant (*P* = 0.037 and 0.002, respectively).

### Model Interpretation

#### Attention Map Visualization

**Figure 2:**
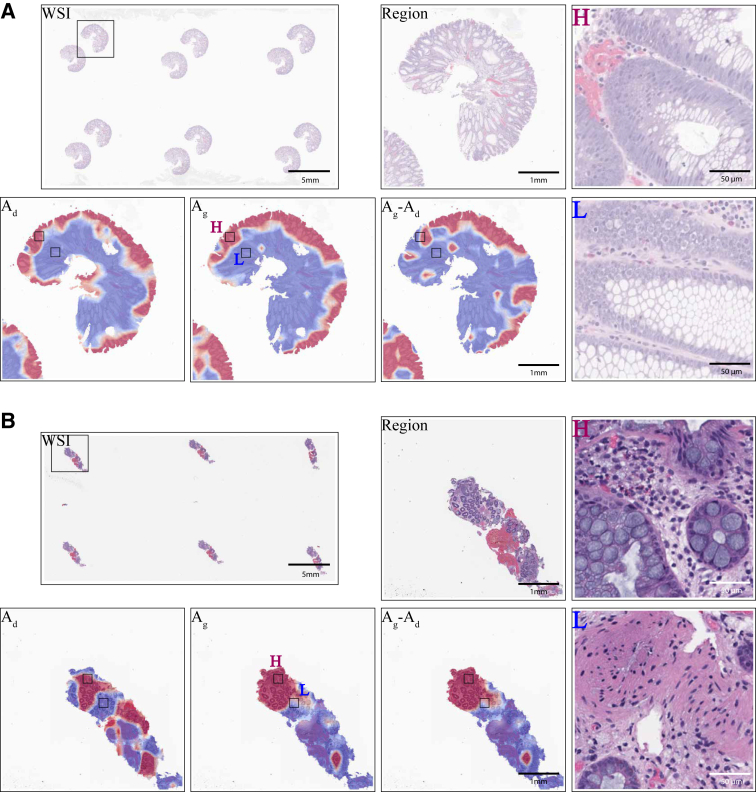
Attention maps for two whole-slide images (WSIs). **A:** High-risk patient. **B:** Low-risk patient. Subpanels: first two: slide/region thumbnails A_d_ and A_g_: attention map generated from direct prediction and guided attention models (the **redder color** indicates higher attention); A_g_-A_d_: attention maps generated by taking the difference between guided attention map and direct attention map (the **redder color** indicates higher attention from the A_g_ model). Detailed patch views correspond to the high attention area (H) and low attention area (L). Scale bars: 5 mm (WSI), 1 mm (Region, A_d_, A_g_, and A_g_-A_d_), and 50 μm (H and L).

The attention maps obtained from the MaskHIT model for two representative WSIs are presented in Figure 2A for a high-risk patient and Figure 2B for a low-risk patient. The visualization reveals that the MaskHIT model tends to focus more on the structures of polyps within the WSIs. Interestingly, the high attention areas appear similar regardless of whether guided fine-tuning methods were used.

The intensity of attention weights from the direct prediction approach and the guided prediction approach was further examined by calculating the difference in attention weights between these two methods. The results are presented in panel “A_g_-A_d_” of Figure 2. The redder color in these panels indicates that the highlighted region received higher attention from the guided prediction model compared with the direct prediction model. This visualization shows that the regions receiving higher attention from the guided prediction model generally align with the regions attended by both the direct prediction model and the guided prediction model. In essence, the guided prediction model exhibited greater confidence in assigning weights to regions that were deemed important for risk prediction.

#### Feature Importance Ranking

**Figure 3:**
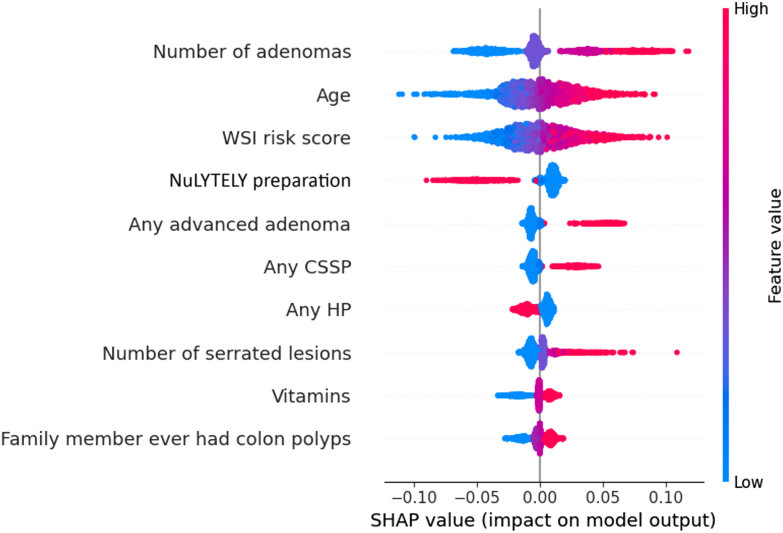
Shapley (SHAP) values of top 10 predictors in the fusion model. CSSP, colorectal sessile serrated polyp; HP, hyperplastic polyp; WSI, whole-slide image.

The top 10 most important features influencing the output of the final fusion model are presented in Figure 3. The most influential feature is the number of adenomas, showing a positive association with 5-year progression risk. Notably, the predicted risk probability from the WSI was ranked as the third most important feature in the fusion model, exceeded only by the number of adenomas and age.

## Discussion

The accurate prediction of future progression risk is crucial for informed decisions regarding follow-up colonoscopy visits. Existing guidelines recommend leveraging polyp characteristics identified in colonoscopy examinations, as well as some personal and family history risk factors, for patient risk stratification to determine the timing of subsequent colonoscopies.^11^ This study sought to advance future progression risk prediction by integrating automatic deep learning–based analysis of WSIs and incorporating CRC-related medical information in a predictive multi-modal pipeline.

Relying exclusively on colonoscopy findings resulted in an average AUC of 0.662. However, by incorporating deep learning–predicted probabilities and information from medical records, a statistically significant improvement in the prediction AUC to 0.672 was observed. This finding underscores the potential of leveraging advanced computational techniques and multi-modal data fusion to enhance progression risk assessment beyond conventional guidelines. Such an approach provides a more robust foundation for personalized and effective follow-up strategies in clinical practice.

To enhance the prediction performance using WSIs, the recently developed model MaskHIT was adopted and adapted. MaskHIT is a transformer-based method that leverages the location information of patches extracted from the entire slide. The unique aspect of the transformer model as a patch-level feature fusion technique lies in its capacity to incorporate spatial details, enabling the deep learning model to capture high-level structural information of the polyps. This approach stands in contrast to commonly used multiple-instance learning approaches, offering a more nuanced and comprehensive representation of the intricate characteristics of colorectal polyps in the predictive model.

In addition, experiments involving a guided prediction approach to improve the transformer model for 5-year progression risk prediction were conducted. As shown in Table 1, predicting 5-year progression risk using WSIs is challenging, as many factors beyond histopathologic features from colonoscopy examinations can influence future progression. Consequently, the MaskHIT model may face difficulties in accurately identifying visual features linked to progression risk in this complex context.

To tackle this challenge, a guided prediction approach was adopted, enabling the transformer model to first predict histopathologic features derived from the colonoscopy examination. Notably, MaskHIT exhibited strong performance in this task, with AUCs exceeding 0.8 and considerably smaller SDs than those in risk prediction tasks. Subsequently, fine-tuning the MaskHIT model for risk prediction led to a statistically significant improvement compared with the direct prediction approach. Interestingly, certain variables, although ineffective at predicting future progression risk independently, also contributed to enhancing MaskHIT’s accuracy in 5-year progression risk prediction.

Attention map visualizations supported the hypothesis, revealing that the guided prediction model assigned greater attention weights to locations relevant for risk prediction (ie, polyps) compared with the direct prediction model. This nuanced approach shows the effectiveness of leveraging the guided prediction approach to enhance the interpretability and performance of deep learning models in the context of 5-year progression risk prediction from WSIs.

Further exploration of various approaches for combining information from colonoscopy examinations, WSIs, and medical records was explored. In general, decision-level fusion produced superior results compared with models combining nonimage features with risk predictions from the slides. Due to the high predictive value of colonoscopy variables, the signal from WSI predictions can be easily overwhelmed by noise in clinical features, a known issue in multi-modal fusion.^77^ However, through the application of decision-level fusion techniques, this challenge can be addressed, resulting in improved outcomes compared with using either modality in isolation, consistent with findings in previous studies.^62^^,^^63^

As future steps, the multi-modal progression risk model will be validated using additional data sets, including prospective and multicenter cohorts. The ultimate goal is to integrate the model-derived risk score into existing guideline-based risk stratification for patients undergoing screening or surveillance colonoscopy. By complementing established criteria, such as polyp type, size, and number, the model could help personalize follow-up intervals, identifying patients who may benefit from earlier surveillance as well as those suitable for extended intervals. The potential health outcomes and cost implications of such an approach could also be assessed through follow-up clinical trials and prospective studies.

## Conclusions

In this study, the integration of the transformer-predicted risk score and additional clinical information resulted in an improvement in the performance of progression risk stratification. Notably, variables describing colonoscopy and microscopy findings of polyps were identified as contributors to enhanced performance in predicting 5-year progression risk using deep learning models. Despite its simplicity in multi-modal fusion, decision-level fusion showed superior performance improvements when combining imaging and nonimaging information. Future research is essential to refine deep learning methods to include more related clinical information and to evaluate the additional benefits of an accurate progression risk stratification in colonoscopy screening programs.

## Disclosure Statement

None declared.
