# Supplementary material for: Improving Colorectal Cancer Screening and Risk Assessment through Predictive Modeling on Medical Images and Records
Source: Am J Pathol. 2025 Oct 16;196(2):493–504. doi: 10.1016/j.ajpath.2025.09.016 (PMC12881281; doi:10.1016/j.ajpath.2025.09.016)
Supplement: Supplemental Table S1 [file mmc1.docx]

**Supplementary Table 1.** Extracted data from NHCR for patients organized in five categories.

| Category | Extracted Data |
| --- | --- |
| Personal history | Age, sex, race and ethnicity, marital status |
|  | Height, weight, body mass index |
|  | Smoking status (duration, cigarettes per day) |
|  | Alcohol consumption (drinks per week) |
|  | Exercise routine, activity level |
|  | Vitamin supplement use (quantity) |
|  | Calcium supplement use (quantity) |
| Medical history | Familial adenomatous polyposis |
|  | Hereditary non-polyposis CRC |
|  | Crohn’s disease or ulcerative colitis |
|  | Constipation or colorectal bleeding |
|  | Aspirin use (dosage) |
| Family history | Relatives diagnosed with CRC (mother/father/sister/brother/child) |
|  | Age of relatives at diagnosis (<50, 50-60, >60) |
|  | Relatives diagnosed with colorectal polyps, familial adenomatous polyposis, hereditary non-polyposis CRC, familial polyposis |
| Previous colonoscopy | Last sigmoidoscopy and colonoscopy time |
|  | Last sigmoidoscopy and colonoscopy outcome |
| Index colonoscopy | Index colonoscopy preparation |
|  | Index colonoscopy findings |
