# Supplementary material for: Improving Colorectal Cancer Screening and Risk Assessment through Predictive Modeling on Medical Images and Records
Source: Am J Pathol. 2025 Oct 16;196(2):493–504. doi: 10.1016/j.ajpath.2025.09.016 (PMC12881281; doi:10.1016/j.ajpath.2025.09.016)
Supplement: Supplemental Table S2 [file mmc2.docx]

**Supplementary Table 2.** Patient description: Personal history.

| Variable | Level | Missing | Grouped by risk | | P-Value |
| --- | --- | --- | --- | --- | --- |
|  |  |  | Low Risk | High Risk |  |
| n |  |  | 1994 | 399 |  |
| Age, mean (SD) |  | 0 | 58.7 (9.8) | 62.0 (8.5) | <0.001 |
| Sex, n (%) | F | 0 | 959 (48.1) | 157 (39.3) | 0.002 |
|  | M |  | 1035 (51.9) | 242 (60.7) |  |
| Marital Status, n (%) | Single | 494 | 113 (7.2) | 20 (6.2) | 0.451 |
|  | Married |  | 1196 (75.8) | 241 (75.1) |  |
|  | Separated |  | 14 (0.9) | 4 (1.2) |  |
|  | Divorced |  | 158 (10.0) | 31 (9.7) |  |
|  | Widowed |  | 53 (3.4) | 18 (5.6) |  |
|  | Living as married |  | 44 (2.8) | 7 (2.2) |  |
| Hispanic, n (%) | Hispanic | 456 | 18 (1.1) | 4 (1.2) | 0.778 |
|  | Not Hispanic |  | 1593 (98.9) | 322 (98.8) |  |
| Race, n (%) | African American | 454 | 5 (0.3) |  | 0.509 |
|  | American Indian |  | 3 (0.2) |  |  |
|  | Asian |  | 6 (0.4) | 2 (0.6) |  |
|  | Caucasian |  | 1548 (95.9) | 314 (96.6) |  |
|  | Multiple |  | 41 (2.5) | 9 (2.8) |  |
|  | Other |  | 11 (0.7) |  |  |
| Exercise, n (%) | No exercise | 466 | 136 (8.5) | 37 (11.4) | 0.029 |
|  | Active daily life |  | 549 (34.2) | 128 (39.5) |  |
|  | 1-5 times/week |  | 760 (47.4) | 126 (38.9) |  |
|  | 5+ times/week |  | 158 (9.9) | 33 (10.2) |  |
| Smoker status, n (%) | Never smoker | 453 | 744 (46.2) | 148 (45.1) | 0.905 |
|  | Former smoker |  | 694 (43.1) | 141 (43.0) |  |
|  | Current smoker |  | 167 (10.4) | 38 (11.6) |  |
|  | Error |  | 7 (0.4) | 1 (0.3) |  |
| Qty smoke, n (%) | Nonsmoker | 506 | 744 (47.5) | 148 (46.1) | 0.949 |
|  | 10 or fewer cigarettes/day |  | 262 (16.7) | 54 (16.8) |  |
|  | 11-20/day |  | 345 (22.0) | 71 (22.1) |  |
|  | 21-30/day |  | 152 (9.7) | 32 (10.0) |  |
|  | 31+/day |  | 63 (4.0) | 16 (5.0) |  |
| Years of smoking, mean (SD) |  | 498 | 10.3 (13.2) | 11.7 (14.8) | 0.123 |
| Weekly alcohol intake, n (%) | 0 alcoholic drinks/week | 471 | 586 (36.7) | 116 (35.6) | 0.423 |
|  | 1-4 alcoholic drinks/week |  | 504 (31.6) | 101 (31.0) |  |
|  | 5-8 alcoholic drinks/week |  | 250 (15.7) | 51 (15.6) |  |
|  | 9-20 alcoholic drinks/week |  | 232 (14.5) | 48 (14.7) |  |
|  | 21+ alcoholic drinks/week |  | 24 (1.5) | 10 (3.1) |  |
| Calcium, n (%) | No calcium use | 1854 | 292 (61.5) | 43 (67.2) | 0.455 |
|  | Calcium use |  | 183 (38.5) | 21 (32.8) |  |
| Vitamins, n (%) | No vitamin use | 1012 | 383 (34.2) | 76 (29.2) | 0.147 |
|  | Vitamin use |  | 738 (65.8) | 184 (70.8) |  |
| Patient weight in inches, mean (SD) |  | 462 | 183.1 (42.4) | 191.2 (43.2) | 0.002 |
| Patient height in pounds, mean (SD) |  | 459 | 67.2 (4.2) | 67.8 (4.5) | 0.015 |
| BMI, median [Q1, Q3] |  | 482 | 27.0 [24.0,31.0] | 28.0 [25.0,32.0] | 0.014 |
