# Supplementary material for: Improving Colorectal Cancer Screening and Risk Assessment through Predictive Modeling on Medical Images and Records
Source: Am J Pathol. 2025 Oct 16;196(2):493–504. doi: 10.1016/j.ajpath.2025.09.016 (PMC12881281; doi:10.1016/j.ajpath.2025.09.016)
Supplement: Supplemental Table S3 [file mmc3.docx]

**Supplementary Table 3.** Patient description: Medical history.

| Variable | Level | Missing | Grouped by risk | | P-Value |
| --- | --- | --- | --- | --- | --- |
|  |  |  | Low risk | High risk |  |
| n |  |  | 1994 | 399 |  |
| History of IBD, n (%) | No | 152 | 1764 (94.3) | 357 (96.5) | 0.111 |
|  | Yes |  | 107 (5.7) | 13 (3.5) |  |
| Genetic syndrome, n (%) | No | 336 | 1711 (99.9) | 344 (100.0) | 1.000 |
|  | Yes |  | 2 (0.1) |  |  |
| Diag exam-change in bowel habits, n (%) | No | 351 | 1691 (99.1) | 335 (99.7) | 0.495 |
|  | Yes |  | 15 (0.9) | 1 (0.3) |  |
| Diag exam-Evaluate GI bleeding, n (%) | No | 351 | 1659 (97.2) | 325 (96.7) | 0.731 |
|  | Yes |  | 47 (2.8) | 11 (3.3) |  |
| Aspirin use, n (%) | No | 527 | 896 (57.5) | 150 (48.5) | 0.004 |
|  | Yes |  | 661 (42.5) | 159 (51.5) |  |
