# Supplementary material for: Improving Colorectal Cancer Screening and Risk Assessment through Predictive Modeling on Medical Images and Records
Source: Am J Pathol. 2025 Oct 16;196(2):493–504. doi: 10.1016/j.ajpath.2025.09.016 (PMC12881281; doi:10.1016/j.ajpath.2025.09.016)
Supplement: Supplemental Table S4 [file mmc4.docx]

**Supplementary Table 4.** Patient description: Family history.

| Variable | Level | Missing | Grouped by risk | | P-Value |
| --- | --- | --- | --- | --- | --- |
|  |  |  | Low risk | High risk |  |
| n |  |  | 1994 | 399 |  |
| Screening exam for family hx of polyp(s), n (%) | No | 351 | 1584 (92.8) | 315 (93.8) | 0.635 |
|  | Yes |  | 122 (7.2) | 21 (6.2) |  |
| Has a biological family member ever had colon polyps, n (%) | No | 447 | 366 (22.6) | 59 (18.0) | 0.129 |
|  | Yes |  | 522 (32.3) | 105 (32.0) |  |
|  | Don’t know |  | 730 (45.1) | 164 (50.0) |  |
| Has a family history of CRC in a first-degree relative, n (%) | No | 417 | 1196 (73.5) | 253 (72.7) | 0.822 |
|  | Yes |  | 432 (26.5) | 95 (27.3) |  |
| Has a family history of CRC in first-degree relative under 50, n (%) | No | 641 | 1369 (94.3) | 279 (93.0) | 0.470 |
|  | Yes |  | 83 (5.7) | 21 (7.0) |  |
| Has a family history of CRC in first-degree relative under 60, n (%) | No | 660 | 1276 (89.4) | 264 (86.6) | 0.190 |
|  | Yes |  | 152 (10.6) | 41 (13.4) |  |
| Patient family member has genetic syndrome, n (%) | No | 344 | 1684 (98.7) | 338 (98.5) | 0.795 |
|  | Yes |  | 22 (1.3) | 5 (1.5) |  |
