# Supplementary material for: Improving Colorectal Cancer Screening and Risk Assessment through Predictive Modeling on Medical Images and Records
Source: Am J Pathol. 2025 Oct 16;196(2):493–504. doi: 10.1016/j.ajpath.2025.09.016 (PMC12881281; doi:10.1016/j.ajpath.2025.09.016)
Supplement: Supplemental Table S5 [file mmc5.docx]

**Supplementary Table 5.** Patient description: Previous colonoscopy.

| Variable | Level | Missing | Grouped by risk | | P-Value |
| --- | --- | --- | --- | --- | --- |
|  |  |  | Low risk | High risk |  |
| n |  |  | 1994 | 399 |  |
| Last sigmoid/colonoscopy Pilot only, n (%) | Never | 1835 | 141 (28.7) | 17 (25.8) | 0.533 |
|  | Within last 12 months |  | 12 (2.4) | 4 (6.1) |  |
|  | 1-4 years ago |  | 114 (23.2) | 17 (25.8) |  |
|  | 5-10 years ago |  | 202 (41.1) | 25 (37.9) |  |
|  | More than 10 years ago |  | 23 (4.7) | 3 (4.5) |  |
| Time since last colonoscopy, n (%) | Never | 982 | 381 (33.1) | 76 (29.2) | 0.254 |
|  | Within last 12 months |  | 39 (3.4) | 10 (3.8) |  |
|  | 1-4 years ago |  | 211 (18.2) | 44 (16.9) |  |
|  | 5-10 years ago |  | 464 (40.3) | 108 (41.5) |  |
|  | More than 10 years ago |  | 56 (4.9) | 22 (8.5) |  |
| Previous colonoscopy findings: polyps, n (%) | No | 979 | 760 (65.9) | 155 (59.4) | 0.055 |
|  | Yes |  | 393 (34.1) | 106 (40.6) |  |
| Previous colonoscopy findings: diverticulosis, n (%) | No | 979 | 1087 (94.3) | 248 (95.0) | 0.747 |
|  | Yes |  | 66 (5.7) | 13 (5.0) |  |
| Previous colonoscopy findings: hemorrhoids, n (%) | No | 979 | 1081 (93.8) | 241 (92.3) | 0.484 |
|  | Yes |  | 72 (6.2) | 20 (7.7) |  |
| Previous colonoscopy findings: other, n (%) | No | 979 | 1108 (96.1) | 253 (96.9) | 0.643 |
|  | Yes |  | 45 (3.9) | 8 (3.1) |  |
| Previous colonoscopy findings: no findings, n (%) | No | 979 | 842 (73.0) | 193 (73.9) | 0.822 |
|  | Yes |  | 311 (27.0) | 68 (26.1) |  |
| Previous colonoscopy results: do not know, n (%) | No | 1872 | 386 (96.0) | 115 (96.6) | 1.000 |
|  | Yes |  | 16 (4.0) | 4 (3.4) |  |
