# Supplementary material for: Improving Colorectal Cancer Screening and Risk Assessment through Predictive Modeling on Medical Images and Records
Source: Am J Pathol. 2025 Oct 16;196(2):493–504. doi: 10.1016/j.ajpath.2025.09.016 (PMC12881281; doi:10.1016/j.ajpath.2025.09.016)
Supplement: Supplemental Table S6 [file mmc6.docx]

**Supplementary Table 6.** Patient description: Index colonoscopy preparation.

| Variable | Level | Missing | Grouped by risk | | P-Value |
| --- | --- | --- | --- | --- | --- |
|  |  |  | Low risk | High risk |  |
| n |  |  | 1994 | 399 |  |
| Procedure - exam preparation quality, n (%) | Excellent | 565 | 486 (31.7) | 74 (25.1) | 0.006 |
|  | Good |  | 906 (59.1) | 203 (68.8) |  |
|  | Fair |  | 141 (9.2) | 18 (6.1) |  |
| Type of preparation: Nulytely, n (%) | No | 104 | 1567 (81.8) | 341 (91.2) | <0.001 |
|  | Yes |  | 348 (18.2) | 33 (8.8) |  |
| Type of preparation: Osmoprep (pills), n (%) | No | 104 | 1915 (100.0) | 374 (100.0) | 1.000 |
| Type of preparation: Half Lytely, n (%) | No | 104 | 1866 (97.4) | 368 (98.4) | 0.359 |
|  | Yes |  | 49 (2.6) | 6 (1.6) |  |
| Type of preparation: Fleet, n (%) | No | 104 | 1909 (99.7) | 373 (99.7) | 1.000 |
|  | Yes |  | 6 (0.3) | 1 (0.3) |  |
| Type of preparation: Other, n (%) | No | 104 | 1907 (99.6) | 372 (99.5) | 0.672 |
|  | Yes |  | 8 (0.4) | 2 (0.5) |  |
