# Supplementary material for: Improving Colorectal Cancer Screening and Risk Assessment through Predictive Modeling on Medical Images and Records
Source: Am J Pathol. 2025 Oct 16;196(2):493–504. doi: 10.1016/j.ajpath.2025.09.016 (PMC12881281; doi:10.1016/j.ajpath.2025.09.016)
Supplement: Supplemental Table S7 [file mmc7.docx]

**Supplementary Table 7.** Patient description: Index colonoscopy exam.

| Variable | Level | Missing | Grouped by risk | | P-Value |
| --- | --- | --- | --- | --- | --- |
|  |  |  | Low risk | High risk |  |
| n |  |  | 1994 | 399 |  |
| Number of records where an adenoma was indicated, median [Q1, Q3] |  | 0 | 1.0 [0.0,1.0] | 1.0 [1.0,3.0] | <0.001 |
| Largest known adenoma size, n (%) | No adenoma | 148 | 599 (31.8) | 70 (19.2) | <0.001 |
|  | <5mm |  | 788 (41.9) | 142 (39.0) |  |
|  | 5-9mm |  | 333 (17.7) | 96 (26.4) |  |
|  | 10-20mm |  | 132 (7.0) | 50 (13.7) |  |
|  | >20mm |  | 29 (1.5) | 6 (1.6) |  |
| Any advanced adenoma at the procedure level, n (%) | No | 186 | 1657 (89.3) | 273 (77.8) | <0.001 |
|  | Yes |  | 199 (10.7) | 78 (22.2) |  |
| Any adenoma at the procedure level, n (%) | No | 0 | 599 (30.0) | 70 (17.5) | <0.001 |
|  | Yes |  | 1395 (70.0) | 329 (82.5) |  |
| Any HP at the procedure level, n (%) | No | 0 | 1182 (59.3) | 274 (68.7) | 0.001 |
|  | Yes |  | 812 (40.7) | 125 (31.3) |  |
| Any SSA/P or TSA w/wo dysplasia at the procedure level, n (%) | No | 0 | 1784 (89.5) | 342 (85.7) | 0.037 |
|  | Yes |  | 210 (10.5) | 57 (14.3) |  |
| Any CSSP at the procedure level, n (%) | No | 164 | 1569 (84.9) | 306 (80.5) | 0.043 |
|  | Yes |  | 280 (15.1) | 74 (19.5) |  |
| Number of serrated lesions identified, median [Q1, Q3] |  | 0 | 0.0 [0.0,1.0] | 0.0 [0.0,1.0] | 0.083 |
| Largest known serrated (missing path/size ignored), n (%) | No serrated polyp | 140 | 1036 (55.3) | 238 (62.5) | <0.001 |
|  | <5mm |  | 585 (31.2) | 75 (19.7) |  |
|  | 5-9mm |  | 171 (9.1) | 48 (12.6) |  |
|  | 10-20mm |  | 71 (3.8) | 17 (4.5) |  |
|  | >20mm |  | 9 (0.5) | 3 (0.8) |  |
| Most advanced adenoma at tissue level, n (%) | No adenoma | 0 | 781 (39.2) | 119 (29.8) | 0.002 |
|  | Tubular adenoma |  | 1159 (58.1) | 263 (65.9) |  |
|  | Tubulovillous adenoma |  | 48 (2.4) | 14 (3.5) |  |
|  | Villous adenoma |  | 6 (0.3) | 3 (0.8) |  |
| Most advanced serrated at tissue level, n (%) | No serrated polyp | 0 | 1353 (67.9) | 299 (74.9) | 0.001 |
|  | Hyperplastic polyp |  | 489 (24.5) | 61 (15.3) |  |
|  | SSP without dysplasia |  | 129 (6.5) | 35 (8.8) |  |
|  | SSP with dysplasia |  | 15 (0.8) | 2 (0.5) |  |
|  | TSA |  | 8 (0.4) | 2 (0.5) |  |
